# Tobacco habituated and non-habituated subjects exhibit different mutational spectrums in head and neck squamous cell carcinoma

**DOI:** 10.1007/s13205-014-0267-0

**Published:** 2014-12-03

**Authors:** Rakesh M. Rawal, Madhvi N. Joshi, Poonam Bhargava, Inayat Shaikh, Aanal S. Pandit, Riddhi P. Patel, Shanaya Patel, Kiran Kothari, Manoj Shah, Akshay Saxena, Snehal B. Bagatharia

**Affiliations:** 1 Gujarat State Biotechnology Mission, Department of Science and Technology, Government of Gujarat, 11th Block, 9th Floor, Udyog Bhavan, Gandhinagar, 382 011 Gujarat India; 2 Gujarat Cancer and Research Institute, Gujarat Cancer Society, Civil Hospital Campus, Asarwa, Ahmedabad, 380 016 Gujarat India

**Keywords:** Head and neck squamous cell carcinoma, Tobacco, Amplicon sequencing, Mutation, Oncogenic transformations

## Abstract

Head and neck squamous cell carcinoma (HNSCC) is the sixth most common non-skin cancer in the world. Tobacco chewing is implicated with most of the cases of HNSCC but this type of cancer is increasing in non-tobacco chewers as well. This study was instigated to provide comprehensive variant and gene-level data in HNSCC subjects of the Indian population and fill the gap in the literature on comparative assessment of gene mutations in cancer subjects with a habit of tobacco and those without any habit using targeted amplicon sequencing. We performed targeted Amplicon sequencing of 409 tumor suppressor genes and oncogenes, frequently mutated across many cancer types, including head and neck. DNA from primary tumor tissues and matched blood was analyzed for HNSCC patients with a habit of tobacco and those without any habit. PDE4DIP, SYNE1, and NOTCH1 emerged as the highly mutated genes in HNSCC. A total of 39 candidate causal variants in 22 unique cancer driver genes were identified in non-habitual (WoH) and habitual (WH) subjects. Comparison of genes from both the subjects, showed seven unique cancer driver genes (KIT, ATM, RNF213, GATA2, DST, RET, CYP2C19) in WoH, while WH showed five (IL7R, PKHD1, MLL3, PTPRD, MAPK8) and 10 genes (SETD2, ATR, CDKN2A, NCOA4, TP53, SYNE1, KAT6B, THBS1, PTPRT, and FGFR3) were common to both subjects. In addition to this NOTCH1, NOTCH2, and NOTCH4 gene were found to be mutated only in habitual subjects. These findings strongly support a causal role for tobacco, acting via PI3K and MAPK pathway inhibition and stimulation of various genes leading to oncogenic transformations in case of tobacco chewers. In case of non-tobacco chewers it appears that mutations in the pathway affecting the squamous epithelial lineage and DNA repair genes lead to HNSCC. Somatic mutation in CYP2C19 gene in the non-habitual subjects suggests that this gene may have a tobacco independent role in development and progression of HNSCC. In addition to sharing high mutation rate, NOTCH gene family was found to be mutated only in habitual sample. Further, presence of mutated genes not earlier reported to be involved in HNSCC, suggest that the Indian sub-continent may have different sets of genes, as compared to other parts of the world, involved in the development and progression of HNSCC.

**Electronic supplementary material:**

The online version of this article (doi:10.1007/s13205-014-0267-0) contains supplementary material, which is available to authorized users.

## Introduction

Head and neck squamous cell carcinoma (HNSCC) is the sixth most common non-skin cancer in the world. More than 600,000 cases are reported per year having a mortality rate of approximately 50 % (Bauman et al. 2012; Ferlay et al. 2010). India contributes to the highest number of incident cases of oral cancer which approximates to 20–30 % of all cancers (http://wwwicmr.nic.in/cancer.pdf). Tobacco chewing, smoking, ill-fitting dentures, poor oral hygiene, syphilis, inadequate diet (lacking in fruits and leafy vegetables), malnutrition, and chronic irritation from rough or broken teeth are common causes implicated to development of oral cancer. Recently, HNSCC has also been associated with HPV virus (Lechner et al. 2013). Vast majority of HNSCC originate due to tobacco chewing, smoking, and/or alcohol consumption (Zeka et al. 2003). However, there are incidences where people who never chewed tobacco or consumed alcohol have developed oral cancer (Subramanian and Govindan 2007). This indicates involvement of genetic susceptibility as well as a complex interaction of genetic and environmental factors in the etiology of oral cancer (Imyanitov et al. 2004).

In the year 2011, the first ever reports of whole exome sequencing of oral cancer patients were revealed (Agrawal et al. 2011; Stransky et al. 2011). Agarwal et al. (2011) sequenced ~18,000 protein coding genes in tumors from 32 patients. A total of 911 candidate somatic mutations were identified in 725 genes of 32 tumors. Stransky et al. (2011) identified 130 coding mutations per tumor. The common finding of both the groups included genes such as TP53, CDKN2A, PIK3CA, and NOTCH1 to be the key players in development of HNSCC. TP53 is the most commonly mutated gene in HNSCC (Agrawal et al. 2011; Stransky et al. 2011; Poeta et al. 2007). All these studies majorly focus on HNSCC patients with a habit of tobacco chewing but the 15 % patients without above-mentioned habits and still found to have development of HNSCC (Rodriguez et al. 2004) remain neglected. Till date there are no reports available on the genetic analysis of these types of patients. Hence, the aim of present study was to analyze genes involved in progression and development of HNSCC among tobacco habituated and non-habituated HNSCC subjects and to analyze differences in the mutational pattern among the two groups using Comprehensive Ampliseq cancer panel of Life Technologies on Ion Torrent Platform followed by bioinformatics analysis.

## Materials and methods

### Materials

DNA was derived from tumors and their matched normal peripheral blood derived mononuclear cells (PBMNC) of four patients. The study comprised three samples of four HNSCC patients using both tumor tissues and matched blood. Sample 1 consisted of tumor tissues of one patient without any habit (WoH); sample 2 consisted of tumor tissue of three patients with tobacco chewing habit (WH). Matched blood of both WH and WoH was taken as control.

Tumor samples from the patients and the matched peripheral blood samples were obtained from the Gujarat Cancer and Research Institute. The demographics and the clinical characteristics of the patient population including age, gender, smoking habit use/exposures were taken as shown in Table 1. Written informed consent was taken from each subject as per institutional guidelines under BioBank Project.

**Table 1:** Demographic, histopathological, and clinical details of patients taken for this study

Subject type	Gender	Age	Ethnic group	Tobacco chewing	Smoking	Alcohol	Tumor site	Histopathological examination
WoH	Male	45	Indian	–	–	–	Right lateral border of the tongue	Well to moderately differentiated squamous cell carcinoma
WH	Male	47	Indian	+	+	+	Left lateral border of the tongue	Well differentiated keratinizing squamous cell carcinoma
WH	Male	51	Indian	+	–	–	Left lateral border of the tongue	Well differentiated squamous cell carcinoma of level grade—I
WH	Male	40	Indian	+	–	–	Left tongue	Moderately differentiated keratinizing squamous cell carcinoma
Control	Matched normal lymphocytes of WoH and WH							

### DNA extraction and sequencing

DNA was isolated from tumor tissue and control blood sample using PureLink^®^ Genomic DNA Extraction Kit (Invitrogen, USA). Qubit^®^ 2.0 Fluorometer (Invitrogen, USA) was used to obtain an accurate quantitation of DNA. DNA quality such as ratio of absorbance at 260/280 and 260/230 nm was measured using Nanophotometer (Imlpen, USA). Library was prepared using Ion Express Plus Fragment library kit (Life technologies, USA). DNA was sheared into blunt-ended fragments by enzymatic lysis using Ion Shear Plus Reagents. The fragments are ligated to Ion Xpress™ Barcode Adapters 1–16, followed by nick-repair to complete the linkage between adapters and DNA inserts. Amplicons were constructed using Ion AmpliSeq™ Comprehensive Cancer Panel (Life technologies, USA) targeting 409 tumor suppressor genes and oncogenes frequently cited and frequently mutated (https://tools.lifetechnologies.com/content/sfs/brochures/CO25560_Ion_AmpliSeq_Comprehensive_Cancer_Panel_Gene_List_final9062012.pdf). All three barcoded libraries were mixed together in one equimolar sample and subsequently processed according to the manufacturer’s protocol using the Ion Xpress Barcode Adaptor kit and sequenced in the Ion Torrent™ Personal Genome Machine™ (PGM™) using an Ion 318™ chip and the Ion Sequencing Kit (300 bp chemistry).

### Data analysis

Reads were mapped onto human hg19 reference genome and quality was assessed by filtering polyclonal and noisy reads, clipping adapters, and low quality reads in Torrent Suite Software 3.4.1 on Ion PGM Torrent Server (http://products.invitrogen.com/ivgn/product/4477685). Barcoded samples were separated and SNVs, insertions and deletions were called using the recommended Torrent Variant Caller 3.6 Plug-in (http://mendel.iontorrent.com/ion-docs/Torrent+Variant+Caller+Plugin.html) with default preset parameters, optimized for ion AmpliSeq comprehensive cancer panel.

Ion Reporter software was used for automated variant annotation derived from public databases (dbSNP, COSMIC, Ensemble, RefSeq) (https://ionreporter.lifetechnologies.com/ir/), which produce classified and annotated variants calls. Ingenuity Variant Analysis software, integerated in the Ion reporter software was used for identification of causal variants and its association with the disease. Low quality variants were filtered out with base call quality <20, to keep only high quality variants. Variants in each sample were compared using online VENNY tool (http://bioinfogp.cnb.csic.es/tools/venny/) to classify into germline and somatic variants.

Non-coding, intronic, and UTR variants were filtered out to keep only coding and splice site variants. Annotated variants found in dbSNP (Sherry et al. 2001) were identified as known variants and variants found in COSMIC database (Bamford et al. 2004) were identified as cancer associated variants. Novel variants were subjected to Polyphen2 (http://genetics.bwh.harvard.edu/pph2/) and SIFT (http://sift.jcvi.org/) software, integrated in ingenuity variant analysis tool for prediction of possible damaging functional effects of variants.

## Results

To accomplish the purpose of the study, three samples from four HNSCC subjects were taken. Sample 1 consisted of tumor tissues of cancer subject without any habit (hereafter referred to as WoH), sample 2 that of subjects with smoking and tobacco chewing habit (hereafter referred to as WH), and sample 3 matched normal lymphocytes of sample 1 and 2 which served as control. All three samples were subjected to targeted amplicon sequencing using Ampliseq Comprehensive cancer panel of Life Technologies on the Ion Torrent Platform.

Reference mapping of reads with human genome (hg19) resulted into 88.6 MB data of 9,61,743 reads for WoH, 119 MB data constituting 12,92,207 reads for WH, and 143 MB of 15,42,354 reads for control as shown in Table 2. An average read length of 110 bp and longest read length of 379 bp was obtained. It was possible to map more than 97 % of reads to target regions with reference genome. High quality reads at 20× coverage were 88 % for WoH, 86 % for WH, and 93 % for control. Mean reads depth coverage was found to be 59.4-fold for WoH, 79.6-folds for WH, and 95.5-fold for control, meeting the requirement of obtaining 100 % variant calling sensitivity.

**Table 2:** Reads mapping details showing number of reads mapped, reads mapped on to the target region, coverage, and coverage depth

Sample	Mapped reads	Reads on target (%)	20 × coverage (%)	Mean coverage depth (fold)
Sample 1 (WoH)	(88.6 MB) 9,61,743 reads	97.35	88.91	59.4883
Sample 2 (WH)	(119 MB) 12,92,207 reads	97.47	86.23	79.6575
Sample 3 (control)	(143 MB) 15,42,354 reads	97.61	93.80	95.5884

### Variant analysis

Further, WoH and WH detected 934 and 959 variations like SNPs, insertions and deletions in 280 and 275 genes, respectively, whereas 1,354 variants in 318 genes detected in control (Table 3). Ti/Tv ratio and dbSNP rate of variants was calculated for statistically validating variant calling quality in each sample. Ratio was calculated for the number of transitions to the number of transversions as it is particularly helpful for assessing the quality of SNP calls (Kalavrezos et al. 2012). Ti/Tv ratios of SNPs were found to be 2.51, 2.61, and 2.52 for WoH, WH, and control samples, respectively, as shown in Table 3. Ti/Tv ratio for novel variants was also calculated and it was found to be 1.0 and 1.4 for WoH and WH, respectively. Accuracy of variant calling was further validated by calculating dbSNP rate of total variants detected in each sample. About 95 % of variants detected in all three samples were already reported in dbSNP database (dbSNP 129) giving an indication of good quality of variant calling.

**Table 3:** Total number of variants and the corresponding genes, percentage of variants in the dbSNP database and there transition/transversion ratio for total and novel (not reported in COSMIC and dbSNP) variants

Sample	Variants	Genes	dbSNP (%)	Ti/Tv total	Ti/Tv novel
Sample 1 (WoH)	934	280	96.90	2.51	1.4
Sample 2 (WH)	959	275	95.50	2.61	1
Sample 3 (control)	1,354	318	94.30	2.52	

### Chromosomal distribution of variants

**Fig. 1:**
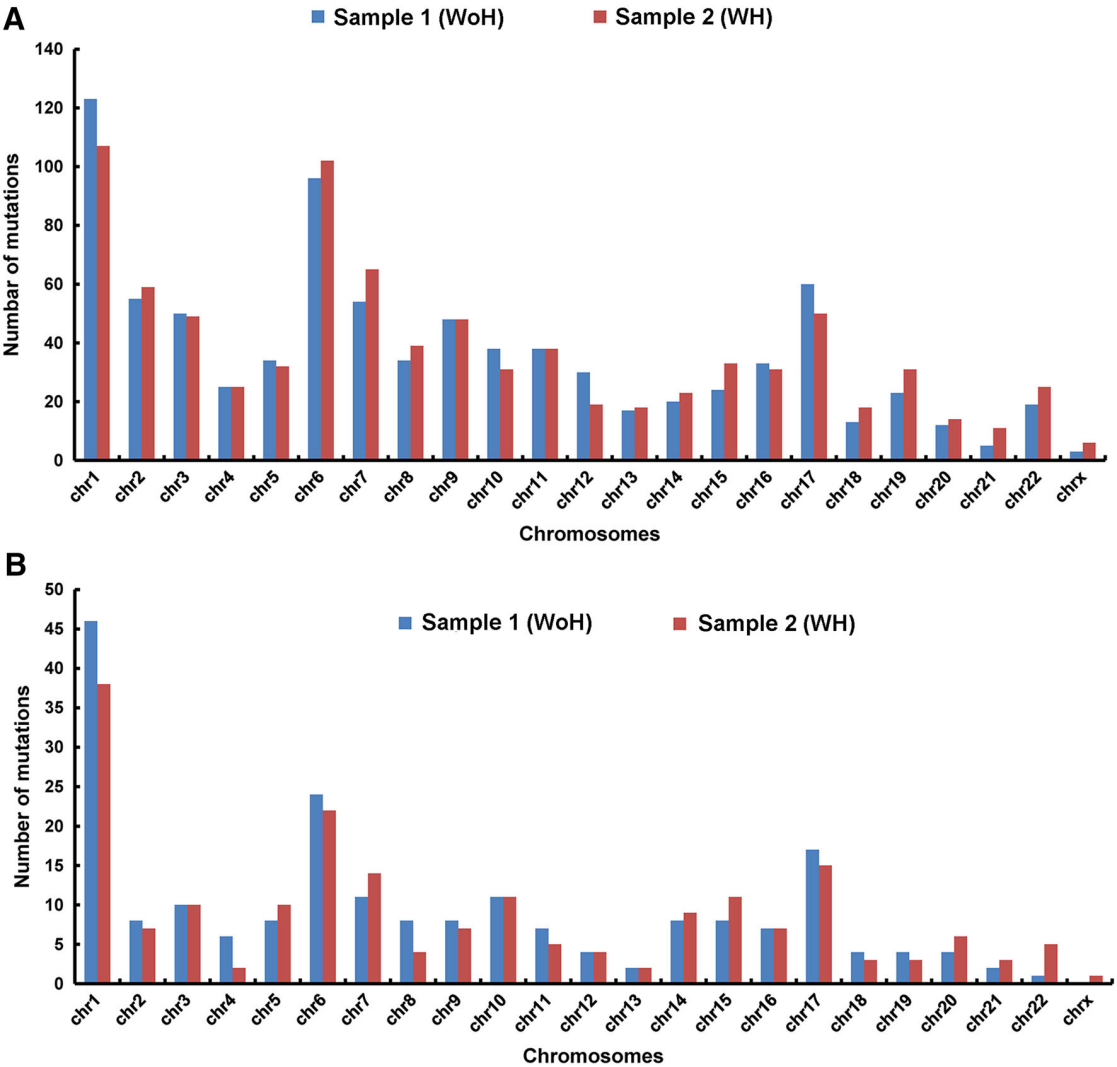
Graph showing variant distribution across all chromosomes (**a**) non-filtered (**b**) filtered (variants with a call quality >20 and only were in exonic and non-synonymous)

Chromosome 1, 6, and 17 were found to have highest number of variants compared to other chromosomes (Fig. 1a). In order to examine whether this pattern was coincidental or consistent, the variants that were present in the exonic regions leading to non-synonymous mutation were only considered. As can be seen in Fig. 1b, the frequency distribution of exonic non-synonymous variants was similar to the non-filtered variants across the chromosomes indicating consistency in both findings.

### Detection of cancer driver variants and genes

**Fig. 2:**
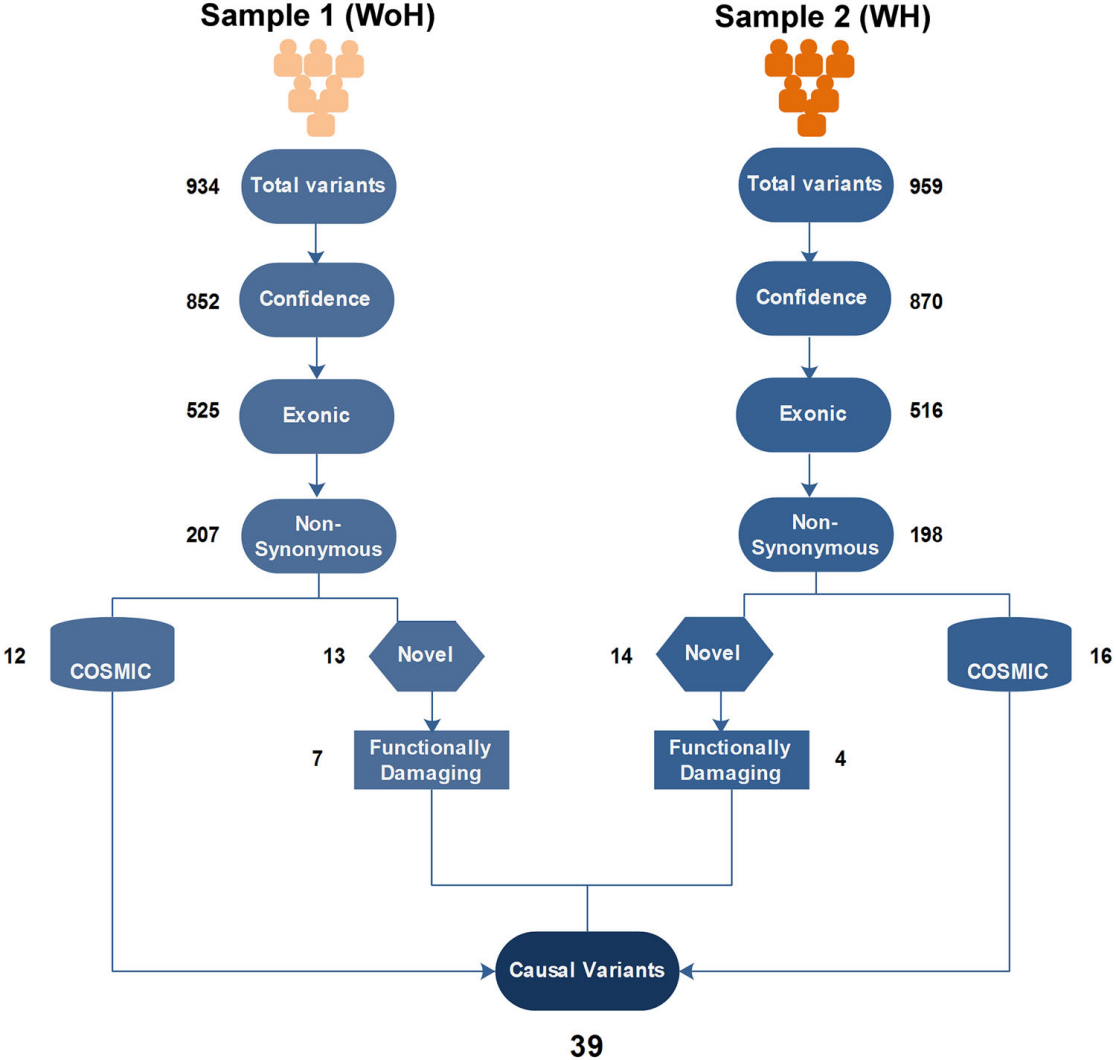
Analysis workflow applied parallel to each sample involving filtering criteria; variants with a call quality >20 were considered as confidence variant. These confidence variants further refined to get variants occuring only in exonic and non-synonymous); functionally damaging (consequences of variation on gene and protein function with the help of bioinformatics) being considered to identify candidate causal variants and genes

A total of 934 and 959 variants were found in WoH and WH, respectively, of which 852 and 870 qualified the confidence criteria (call quality >20). Further, 525 variants from WoH and 516 from WH, corresponded to exonic regions, of which 207 and 198, respectively, were non-synonymous (Fig. 2).

**Fig. 3:**
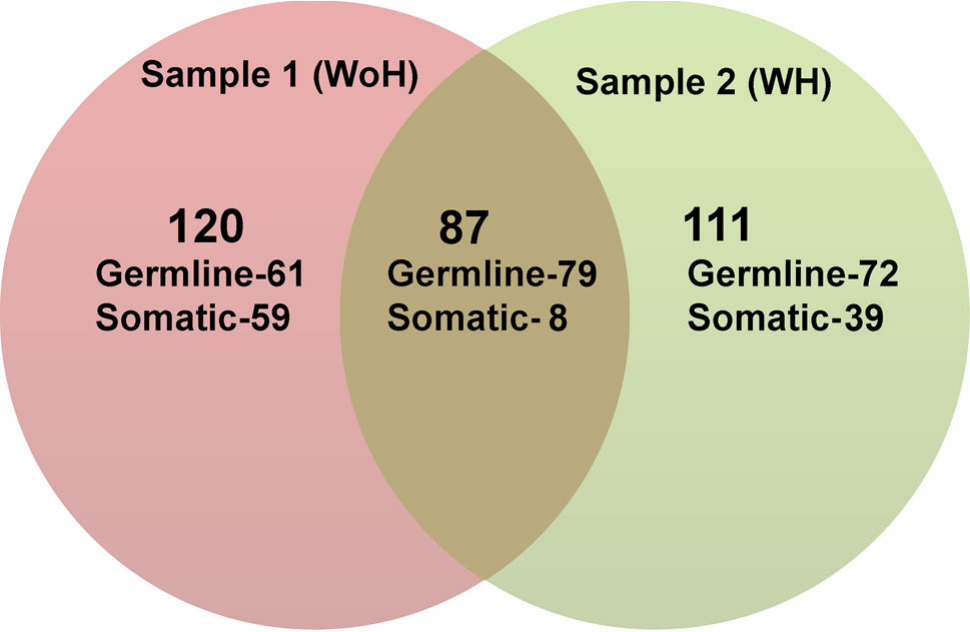
Venn diagram depicting a comparative assessment of non-synonymous variants from both the samples. Sample 1 (*red*) and Sample 2 (*green*). Somatic and germline variants in each sample identified by comparing with its corresponding matched control. Variants from each samples found in matched control, were referred as germline whereas rest (sample specific variants) are considered as somatic. Each sample depicting non-habitual, habit specific (shown in *green*), and non-specific (shown in *brown*) somatic and germline variants

Of the non-synonymous variants 87 were common and 120 and 111 were unique in WoH and WH samples, respectively (supplementary). In order to classify these variants into tissue specific (somatic) and germline, variants from control sample were considered. Out of 87 common variants in both the samples 79 variants were germline and eight were somatic (Fig. 3). WoH showed 61 germline and 59 somatic variants, whereas WH harbored 72 germline and 39 somatic variants.

These variants were then annotated against dbSNP and COSMIC databases. 194 variants from WoH and 184 of WH were found to be already reported in dbSNP database (dbSNP 129). Of these, a total of 28 variants were also reported in COSMIC (Table 4). However, 27 variants could not be found in either dbSNP or COSMIC and hereafter referred to as novel. Out of the 27 novel variants, 11 were predicted to be functionally damaging as depicted from SIFT and Polyphen2. Therefore, total 39 (28 COSMIC and 11 novel functionally damaging) variants were identified as causative cancer driver variants in both samples (Table 4). WoH and WH showed 19 and 20 causative cancer driver variants, respectively. Comparative assessment revealed nine common variants in both samples, while 10 and 11 unique variants in WoH and WH, respectively.

**Table 4:** Details of cancer driver variants (present in COSMIC and novel functionally damaging) in habitual and non-habitual sample

Subject	COSMIC/novel	Chromosome	Genomic position	Reference allele	Sample allele	Read depth	Variation type	Cytoband	Gene region	Gene symbol	Protein variant	Sample-genotype	Somatic/germline
Habitual (WH)	Novel functionally damaging	4	1,808,286	G	A	55	SNV	p16.3	Exonic	FGFR3	p.V682I; p.V684I; p.V570I	Het	Somatic
		10	49,634,537	A	C	45	SNV	q11.22	Exonic	MAPK8	p.E329A	Het	Somatic
		15	39,884,882	G	T	68	SNV	q14	Exonic	THBS1	p.Q882H	Het	Germline
		20	41,420,019	T	C	108	SNV	q12	Exonic	PTPRT	p.H101R	Het	Germline
	COSMIC	10	51,568,378	T	G	133	SNV	q11.23	Intronic; Exonic	NCOA4	p.F8V	Hom	Somatic
		17	7,577,538	C	T	111	SNV	p13.1	Exonic	TP53	p.R209Q; p.R116Q; p.R248Q	Het	Somatic
		3	47,125,385	G	A	29	SNV	p21.31	Exonic	SETD2	p.P1962L	Het	Germline
		3	142,178,144	C	T	104	SNV	q23	Exonic	ATR	p.R2425Q	Het	Germline
		3	142,281,612	A	G	119	SNV	q23	Exonic	ATR	p.M211T	Het	Germline
		5	35,861,068	T	C	163	SNV	p13.2	Exonic	IL7R	p.I66T	Het	Germline
		5	35,871,190	G	A	152	SNV	p13.2	Exonic	IL7R	p.V138I	Het	Germline
		6	51,910,905	T	C	160	SNV	p12.2	Exonic	PKHD1	p.N830S	Het	Germline
		6	152,665,261	C	A	100	SNV	q25.2	Exonic	SYNE1	p.E3989D; p.E4060D	Het	Germline
		7	151,882,672	C	A	79	SNV	q36.1	Exonic	MLL3	p.A1685S	Het	Germline
		7	151,962,265	C	T	855	SNV	q36.1	Exonic	MLL3	p.D348N	Het	Germline
		9	8,518,052	G	C	103	SNV	p24.1	Exonic	PTPRD	p.Q447E; p.Q444E; p.Q441E; p.Q437E	Het	Germline
		9	21,968,199	C	G	57	SNV	p21.3	3′UTR	CDKN2A		Hom	Germline
		10	76,781,908		GAGGATGAAGAGGAGGAAGAAGAG	126	Insertion	q22.2	Exonic	KAT6B	p.914_915insEDEEEEEE; p.805_806insEDEEEEEE; p.1097_1098insEDEEEEEE	Het	Germline
		15	39,880,822	A	G	204	SNV	q14	Exonic	THBS1	p.T523A	Het	Germline
		17	7,579,472	G	C	96	SNV	p13.1	Promoter; Exonic	TP53	p.P72R; p.P33R	Het	Germline
Non-Habitual (WoH)	COSMIC	6	152,665,261	C	A	33	SNV	q25.2	Exonic	SYNE1	p.E3989D; p.E4060D	Hom	Somatic
		10	51,568,378	T	G	97	SNV	q11.23	Intronic; Exonic	NCOA4	p.F8V	Hom	Somatic
		10	96,540,410	G	A	103	SNV	q23.33	Exonic	CYP2C19	p.W212*	Het	Somatic
		17	78,302,157	C	A	53	SNV	q25.3	Exonic	RNF213	p.Q1133K	Hom	Somatic
		17	78,319,136	G	A	103	SNV	q25.3	Exonic	RNF213	p.S2334N	Hom	Somatic
		3	47,125,385	G	A	71	SNV	p21.31	Exonic	SETD2	p.P1962L	Het	Germline
		3	142,281,612	A	G	171	SNV	q23	Exonic	ATR	p.M211T	Het	Germline
		4	55,593,464	A	C	42	SNV	q12	Exonic	KIT	p.M541L; p.M537L	Het	Germline
		9	21,968,199	C	G	65	SNV	p21.3	3′UTR	CDKN2A		Hom	Germline
		10	76,781,908		GAGGATGAAGAGGAGGAAGAAGAG	69	Insertion	q22.2	Exonic	KAT6B	p.914_915insEDEEEEEE; p.805_806insEDEEEEEE; p.1097_1098insEDEEEEEE	Het	Germline
		11	108,175,462	G	A	111	SNV	q22.3	Exonic	ATM	p.D1853N	Het	Germline
		17	7,579,472	G	C	60	SNV	p13.1	Promoter; Exonic	TP53	p.P72R; p.P33R	Het	Germline
	Novel functionally damaging	4	1,808,286	G	A	34	SNV	p16.3	Exonic	FGFR3	p.V682I; p.V684I; p.V570I	Het	Somatic
		3	128,202,753	G	A	46	SNV	q21.3	Exonic	GATA2	p.H323Y	Het	Germline
		6	56,325,048	G	A	40	SNV	p12.1	Exonic	DST	p.T5045M	Het	Germline
		6	152,638,007	G	A	42	SNV	q25.2	Exonic	SYNE1	p.R5563W; p.R5492W	Het	Germline
		10	43,615,078	G	T	72	SNV	q11.21	Exonic	RET	p.G831V	Het	Germline
		15	39,884,882	G	T	28	SNV	q14	Exonic	THBS1	p.Q882H	Het	Germline
		20	41,420,019	T	C	55	SNV	q12	Exonic	PTPRT	p.H101R	Het	Germline

The details include chromosome number, position, reference allele, sample allele, Read depth, variation type, cytoband loci, gene region, gene involved, protein variant, genotype and Somatic or Germline

**Fig. 4:**
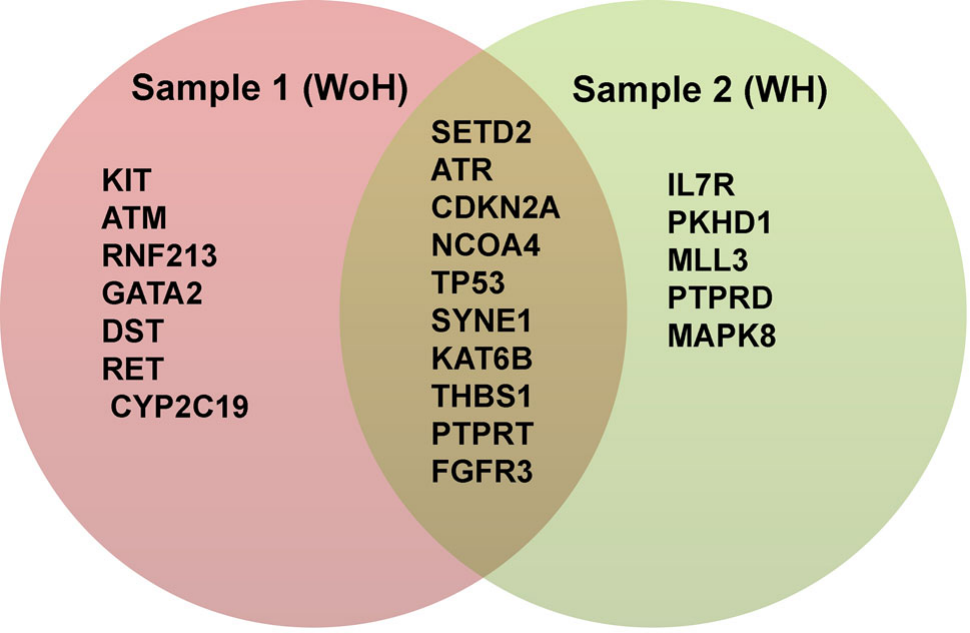
Venn diagram showing cancer driver genes in each sample. Genes in *red* and *green* background are unique to non-habitual and habitual sample, respectively; genes in *gray* background common to both the samples

Novel variants found in COSMIC involved 17 genes in case of WoH and 15 in case of WH. A total of 22 unique genes were found in both WoH and WH subjects. Of which, 7 (KIT, ATM, RNF213, GATA2, DST, RET, and CYP2C19) were unique in WoH, 5 (IL7R, PKHD1, MLL3, PTPRD, and MAPK8) in WH. Genes found to be common were 10 (SETD2, ATR, CDKN2A, NCOA4, TP53, SYNE1, KAT6B, THBS1, PTPRT, and FGFR3) in both samples as shown in Fig. 4. Comparison of candidate variants and genes with two of the most comprehensive studies in HNSCC, showed no similarity at the variant level. Further six genes GATA2, IL7R, KAT6B, MAPK8, NCOA4, and SETD2 were commonly mutated in all the three datasets i.e., Agrawal, Stranskey, and this study.

### Assessment of mutation distribution at gene level

Average percentage mutation rate distribution shared by all detected cancer panel genes was 0.32 %. While, mutations shared by cancer driver genes was 0.62 % in all three samples, which is almost double the average mutations harbored by all panel genes. PDE4DIP gene emerged as most mutated gene with a mutation rate of 5.86 % followed by SYNE1 with a mutation rate (3.07 %) from both the samples. Other genes exhibiting highest mutation included PKHD1, LRP1B, NOTCH1, FN1, and RNF213 sharing 1.97, 1.50, 1.33, 1.16, and 1.16 % mutations, respectively, in both samples.

## Discussion

Onset and progression of cancer has been always dynamically linked with various reasons involving physical/or chemical agents, genetic v/s environment and hence is a very complex disease. In the present study, an attempt has been made to understand the genetic difference in the two different types of HNSCC subjects in the backdrop of habit (WH) and no habit (WoH). The results obtained showed distinct pattern with reference to association of genes in both set of subjects. Number of mutations observed in subjects with a habit of tobacco was more compared to number in subjects with no such habit. Association of tobacoo, alcohol, and their products in development of various cancers specially HNSCC is well studied and tobacco has several carcinogens such as Benzo[a]pyrene diol epoxide, including benzo[a]pyrene, tobacco-specific nitrosamines such as nitrosonornicotine, and reactive aldehydes such as formaldehyde etc. Some of these carcinogens like Benzo[a]pyrene diol epoxide is also linked with induction of genetic damage in the DNA (Denissenko et al. 1996).

As can be seen from Fig. 1a, b, more number of mutations were distributed on chromosomes 1, 6, and 17. Chromosome 1 being the largest chromosome with highest number of genes attributed to highest number of mutations. Chromosome six harbors the major histocompatibility complex (Abdulla et al. 1996), and chromosome 17 genes involved in protein synthesis and gene regulation. Although the mutations in the chromosomes are generally considered as random events but there are certain regions which are more prone to mutation such as repetitive regions. In case of chromosome 17, percentage of low copy repeats is more than 23 % and hence is more prone to mutations (Stankiewicz et al. 2003; Clancy and Shaw 2008). Further, previously reported microsatellite instability and loss of heterozygosity during some cancers on chromosomes 6 and 17 (Roy et al. 2001; Amos et al. 2010; Lan et al. 2012) support the data obtained. The most promising variants were found in the q arm of these chromosomes. Highest mutations occurred in the 1q21 which harbor the PDE4DIP gene, 6q25 harbor the SYNE1 gene, and 17q25 harbor RNF13. Of these PDE4DIP, SYNE1, and NOTCH1 gene emerged as the genes sharing highest mutations. This clearly indicates possible role of PDE4DIP and SYNE1 genes in HNSCC in both the type of subjects i.e., with or without habits of tobacco; whereas NOTCH gene family which are mutated only in habitual subjects suggests its tobacco-dependent role.

Despite a large number of studies on genetic analysis of HNSCC (Bauman et al. 2012; Agrawal et al. 2011; Stransky et al. 2011; Poeta et al. 2007), there are no reports regarding involvement of PDE4DIP in HNSCC. Further, studies on SYNE1 in case of HNSCC are less characterized (Stransky et al. 2011) in certain populations. This could be because most of the studies are from the developed countries with different races where the genetic makeup, socio-economic background, food habits are quite different from those of India. This explanation also holds true for non-overlap of variants found in this study and those done in the Western countries.

The protein encoded by PDE4DIP gene serves to anchor phosphodiesterase 4D to the Golgi/centrosome region of the cell. The mutations have also been previously identified as diagnostic marker in esophageal squamous cell carcinoma (SCC) (Shimada et al. 2007). SYNE1 gene is known to be involved in nuclear polarity and spindle orientation (Luke et al. 2008) that function upstream of NOTCH1 signaling in the squamous cell differentiation pathway (Williams et al. 2011). Hence, mutations in both genes may lead to alterations in pathways of squamous cell differentiation ultimately leading to onset of HNSCC.

Apart from SYNE1 and PKHD1 genes which were found to be maximally mutated to 22 other genes were identified as cancer driver genes which harbor 39 variants. Out of which, 10 genes were common in both the samples while 7 and 5 genes unique to WoH and WH, respectively. These genes include NCOA4, SYNE1, SETD2, ATR, CDKN2A, KAT6B, TP53, FGFR3, THBS1, and PTPRT common to both the samples whereas CYP2C19, RNF213, KIT, ATM, GATA2, DST, and RET genes unique to WoH and IL7R, PKHD1, MLL3, PTPRD, and MAPK8 unique to WH.

Of the genes commonly found in both the samples, PTPRT has been previously reported to be frequently mutated in cancer patients (Zhao et al. 2010). It supposedly regulates paxillin level which in turn affects regulation of several signaling pathways. Paxillin also regulates expression of Nesprin-3 which influences actin diamines. Since any disturbance in the regulation of actin dynamics may lead to the disformation of the core cytoskeleton PTPRT may be having a role in the disfigurement during HNSCC. Further, CDKN2A, ATR, THBS1, NCOA4, PTPRD, TP53, and ATM are known tumor suppressor genes (McLendon et al. 2008; Pardali and Moustakas 2007; Painter and Young 1980; Ligr et al. 2010; Bose et al. 2009; Kohno et al. 2010). Alteration of tumor-suppressor genes may allow cellular proliferation to continue with unregulated and autonomous, self-sufficient growth (Field et al. 1993) thereby initiating cancerous growth. Mutations in TP53 are shown to exist in over 50 % of HNSCC lesions (Argiris et al. 2008) and have been shown to be one of the most common genetic abnormalities in human cancers (Field et al. 1993). NCOA4 expression studies have reported its role in oral cancer and recognized as candidate serum markers for oral squamous cell carcinomas (OSCC) (Kollara and Brown 2012). CDKN2A binds to CDK4 and CDK6 and suppresses proliferation by inhibiting cells progressing from G1 into S phase (Liggett and Sidransky 1998), and has also been recognized as an early event in the progression of pre-malignant lesions leading to HNSCC (Schwarz et al. 2008). PTPRD is previously reported candidate tumor suppressor gene in lung cancer (Kohno et al. 2010).

Among the unique genes associated with WoH, mutation in GATA2 has been reported to be involved in dysplasia (Fadilah et al. 2002). Similarly, DST gene is a protein coding gene required for anchoring either intermediate filaments to the actin cytoskeleton in neural and muscle cells (Dalpe et al. 1998) or keratin-containing intermediate filaments to hemidesmosomes in epithelial cells. Since HNSCC involves transformation of squamous epithelial lineage, presence of mutation in genes disrupting epithelia leading to development of cancer is justified. DNA damage sensor and control genes (ATM and ATR), (Goldgar et al. 2011; Tanaka et al. 2012) were also found to harbor mutation thereby disrupting the DNA damage control pathway. RNF213 gene is involved in the pathogenesis of moyamoya disease which has been reported to be both congenital and acquired (Sonobe et al. 2014; Mineharu et al. 2013). Actual role of this gene in HNSCC is not known but it may have a probable role in angiogenesis reported in advanced stages of cancer.

One very interesting mutation was found in the RET gene which suggests a genetic basis for development of HNSCC in WoH subjects. Mutations in RET gene are reportedly germline and has function of signaling pathway of tyrosine kinase. Further, it is also reported that specific mutations in this gene leads to development of specific tumors. For example, single point mutation at codon 918 results in Multiple Endocrine Neoplasia (Mulligan et al. 1995) and that in codon 664 lead to small cell lung carcinoma (Futami et al. 1994). In this study, we report a novel germline mutation of codon 831 which converts glycine to valine (Table 4). Functionally this mutation affects the tyrosine kinase domain of the protein thereby probably disturbing the substrate specificity. Further validation of the mutations in this gene with more subjects may provide substance to suggest a genetic basis in occurrence of HNSCC in WoH subjects.

Another important gene uniquely mutated in WoH was CYP2C19 gene. This gene is responsible for xenobiotic metabolism (Marzo and Balant 1996). It is also involved in the activation of different carcinogen and/or inactivation of cancer-related drugs (Antona and Sundberg 2006). Thus it may be suggested that mutations in this gene may have role in the effect of drug during therapy of HNSCC. Thus mutations in this gene may be assessed before starting a cancer treatment for effective drug effect.

On the other hand, the unique mutated genes in WH subjects i.e., sample 2 included IL7R, PKHD1, MLL3, PTPRD, and MAPK8. IL7R is reported in childhood T-cell acute lymphoblastic leukemia, promotes cell transformation and tumor formation (Zenatti et al. 2011). Of these, MLL3 is a known tumor suppressor gene (Lee et al. 2009) which contributes to chromatin remodeling and transcriptional regulation in cancers (Fujimoto et al. 2012; Zang et al. 2012). It also has a key role in PI3K pathway (intracellular signaling pathway important in apoptosis), activation of which is a hallmark of a variety of malignancies, including melanoma and high-grade astrocytomas (McLendon et al. 2008; Lui et al. 2013). Additionally both IL7R and MLL3 reportedly affect histone acetyl binding (Chowdhury and Sen 2003; Li et al. 2013). Histone acetylation and deacytylation is not only important for regulation of genes involved in the genesis of cancer but also regulation of angiogenesis that permits increased tumor growth as well as the regulation of adhesion, cell migration, and invasion required for metastasis. Further, PTPRD is a known tumor suppressor for lung cancer (Kohno et al. 2010); glioblastoma (Veeriaha et al. 2009). It is a central feature in signaling cascades such as signal transducers and activators of transcription (STAT3) pathway (Veeriaha et al. 2009) involved in oncogenesis (Lui et al. 2013). Further, MAPK8 gene is known to be stimulated by tobacco smoke thereby disturbing the MAPK pathway, an important targetable pathway in HNSCC (Lui et al. 2013). PKHD1 induces cell apoptosis through PI3K and NF-κB pathways (Sun et al. 2011).

Thus analysis of the exclusive gene mutations in the WoH subject indicates that it is the mutation in epithelial transformation and DNA damage repair genes which in turn lead to HNSCC. In view of the RNF13 gene, the study also suggests genetic origin of HNSCC. In contrast to this in case of WH subjects most of the mutations are directly in the oncogeneic genes probably because tobacco itself is carcinogenic and no hereditary basis was found.

## Conclusion

Thus one can suggest that in case of non-tobacco chewers, mutation in the genes involved in squamous epithelial development and DNA repair genes lead to HNSCC, while it is the carcinogen tobacco in chewers which leads to the stimulation of various oncogenes and tumor suppressor genes leading to HNSCC. Further, presence of novel mutations not reported earlier in HNSCC patients also suggest that Indian sub-continent may have different sets of genes, as compared to other parts of the world, involved in the development and progression of HNSCC. However, we need to strengthen the statements by further sequencing of the subset of the shortlisted genes from each sample on a larger sample patient’s population.

## Electronic supplementary material

Below is the link to the electronic supplementary material.

Supplementary material 1 (XLSX 48 kb)
